# XVII Reunión Post-ECTRIMS: Revisión de las Novedades Presentadas en el Congreso ECTRIMS 2024 (Parte I)

**DOI:** 10.31083/RN39227

**Published:** 2026-01-21

**Authors:** Óscar Fernández, Adrián Arés, Eduardo Agüera, Yolanda Aladro, Ana Alonso, Rafael Arroyo, Luis Brieva, Carmen Calles, Ana Belén Caminero, Tamara Castillo-Triviño, Lucienne Costa-Frossard, Sara Eichau, Miguel Ángel Hernández, Lamberto Landete, Miguel Llaneza, Sara Llufriu, José E. Meca-Lallana, Virginia Meca-Lallana, Ester Moral, Celia Oreja-Guevara, José María Prieto, Lucía Romero-Pinel, Andreu Vilaseca, Alfredo Rodríguez-Antigüedad

**Affiliations:** ^1^Departamento de Farmacología, Facultad de Medicina, Instituto de Investigación Biomédica de Málaga (IBIMA), Hospital Universitario Regional de Málaga, Universidad de Málaga, 29010 Málaga, España; ^2^Departamento de Neurología, Complejo Asistencial Universitario de León, 24008 León, España; ^3^Servicio de Neurología, Hospital Reina Sofía, 14004 Córdoba, España; ^4^Servicio de Neurología, Hospital Universitario de Getafe, 28905 Madrid, España; ^5^Unidad de Esclerosis Múltiple, Servicio de Neurología, Hospital Regional Universitario de Málaga, 29010 Málaga, España; ^6^Servicio de Neurología, Hospital Universitario Quirónsalud, 28223 Madrid, España; ^7^Departamento de Medicina, Universitat de Lleida, Hospital Universitari Arnau de Vilanova, 25198 Lleida, España; ^8^Servicio de Neurología, Hospital Universitario Son Espases, 07120 Palma de Mallorca, España; ^9^Departamento de Neurología, Complejo Asistencial de Ávila, 05071 Ávila, España; ^10^Servicio de Neurología, Hospital Universitario Donostia, Grupo de Neuroinmunología, IIS Biogipuzkoa, 20014 Donostia, España; ^11^CSUR de Esclerosis Múltiple, Hospital Ramón y Cajal, 28034 Madrid, España; ^12^Servicio de Neurología, Hospital Universitario Virgen Macarena, 41009 Sevilla, España; ^13^Servicio de Neurología, Hospital Nuestra Señora de Candelaria, 38010 Santa Cruz de Tenerife, España; ^14^Servicio de Neurología, Hospital Universitario Doctor Peset, 46017 Valencia, España; ^15^Servicio de Neurología, Hospital Universitario Central de Asturias, 33011 Oviedo, España; ^16^Unidad de Neuroinmunología y Esclerosis Múltiple, Hospital Clínic de Barcelona e IDIBAPS, 08036 Barcelona, España; ^17^Unidad de Neuroinmunología Clínica y CSUR Esclerosis Múltiple, Servicio de Neurología, Hospital Clínico Universitario Virgen de la Arrixaca (IMIB-Arrixaca), Cátedra de Neuroinmunología Clínica y Esclerosis Múltiple, Universidad Católica San Antonio (UCAM), 30120 Murcia, España; ^18^Servicio de Neurología, Hospital Universitario de la Princesa, 28006 Madrid, España; ^19^Servicio de Neurología, Complejo Hospitalario Universitario Moisès Broggi, 08970 Barcelona, España; ^20^Servicio de Neurología, Hospital Clínico San Carlos, IdISSC; Departamento de Medicina, Facultad de Medicina, Universidad Complutense de Madrid (UCM), 28040 Madrid, España; ^21^Servicio de Neurología, Instituto de Investigación Sanitaria de Santiago de Compostela (IDIS), 15706 Santiago de Compostela, España; ^22^Departamento de Neurologia, Hospital Universitari de Bellvitge-IDIBELL, 08908 L’Hospitalet de Llobregat, España; ^23^Servei de Neurologia, Hospital Universitario Vall d’Hebron, CEMCAT, 08035 Barcelona, España; ^24^Servicio de Neurología, Hospital Universitario Cruces, 48903 Barakaldo, España

**Keywords:** ECTRIMS, esclerosis múltiple, post-ECTRIMS, ECTRIMS, multiple sclerosis, post-ECTRIMS

## Abstract

**Introducción:**

La XVII edición de la reunión post-Comité Europeo para el Tratamiento y la Investigación de la Esclerosis Múltiple (ECTRIMS) se celebró los días 4 y 5 de octubre de 2024 en Madrid. Este evento congregó a neurólogos españoles especializados en esclerosis múltiple (EM), quienes presentaron un resumen de los avances más relevantes discutidos en el congreso ECTRIMS, celebrado días antes en Copenhague.

**Objetivo:**

Presentar las novedades sobre neurodegeneración y progresión, la fase prodrómica y el diagnóstico, el uso clínico de los biomarcadores y de la neuroimagen, así como el papel actual de los resultados informados por los pacientes y la monitorización digital. También se resume lo más destacado en cuanto al riesgo de infecciones y de comorbilidades en la EM.

**Desarrollo y Conclusiones:**

En lesiones activas de EM no hay correlación entre el fenotipo de célula mieloide y la remielinización, y los astrocitos de memoria, regulados por el gen *CLEC16A*, están presentes en lesiones crónicas activas. La atrofia de sustancia gris se asocia con discapacidad y progresión independiente de los brotes, mientras que la atrofia medular cervical predice el pronóstico de formas progresivas y adelanta el diagnóstico. El uso de recursos sanitarios aumenta en los años previos al primer evento desmielinizante, y los síntomas prodrómicos, aunque muy variados, resultan útiles para identificar factores de riesgo de la enfermedad. Los nuevos criterios de McDonald facilitarán el diagnóstico de EM en pacientes con síndrome radiológico aislado. La proteína acídica fibrilar glial complementa a los neurofilamentos y ambos biomarcadores podrían usarse en breve de forma estandarizada en la práctica clínica; las lesiones con borde paramagnético y de expansión lenta son marcadores de imagen prometedores. En otra línea, los resultados en salud informados por los pacientes son valiosos, aunque enfrentan sesgos de selección y la necesidad de establecer límites en su uso. Por último, el riesgo de infecciones aumenta antes del diagnóstico y puede agravarse con ciertos tratamientos. La comorbilidad en EM debe manejarse como parte integral del manejo de la enfermedad.

## Abstract (en)

**Introduction:**

The XVII edition of the post-European Committee for Treatment and Research in Multiple Sclerosis (ECTRIMS) meeting was held on 4–5 October 2024 in Madrid. This event was attended by Spanish neurologists specialized in multiple sclerosis (MS), who presented a summary of the most relevant advances discussed at the ECTRIMS congress, held days before in Copenhagen.

**Aim:**

To present new developments in neurodegeneration and progression, the prodromal phase and diagnosis, the clinical use of biomarkers and neuroimaging, as well as the current role of patient-reported outcomes and digital monitoring. Highlights on the risk of infections and comorbidities in MS are also summarized.

**Content and Conclusions:**

In active MS lesions, there is no correlation between the myeloid cell phenotype and remyelination, while memory astrocytes, regulated by the CLEC16A gene, are present in chronic active lesions. Gray matter atrophy is associated with disability and progression independent of relapses, whereas cervical spinal cord atrophy predicts the prognosis of progressive forms and may lead to earlier diagnosis. Healthcare resource utilization increases in the years preceding the first demyelinating event, and although prodromal symptoms are highly variable, they are useful in identifying risk factors for the disease. The new McDonald criteria will facilitate the diagnosis of MS in patients with a radiologically isolated syndrome. Glial fibrillary acidic protein complements neurofilaments, and both biomarkers could soon be standardized for use in clinical practice; paramagnetic rim lesions and slowly expanding lesions are promising imaging markers. In another area, patient-reported health outcomes are valuable, although they are subject to selection bias and the need to define boundaries for their use. Finally, the risk of infections increases before diagnosis and may worsen with certain treatments. Comorbidities in MS should be managed as an integral part of disease management.

## 1. Introducción

Los días 4 y 5 de octubre de 2024 se celebró en Madrid la XVII edición de la reunión post-ECTRIMS. Como cada año, varios neurólogos expertos en esclerosis múltiple (EM) en España presentaron un resumen de las principales novedades del congreso ECTRIMS celebrado en Copenhague unos días antes. El presente artículo resume las novedades en neurodegeneración y progresión, fase prodrómica y diagnóstico, y la utilidad clínica de los biomarcadores y la neuroimagen. Asimismo, se presentan las perspectivas de las medidas de resultados informados por los pacientes, y las implicaciones clínicas de la monitorización digital. Se destaca también el riesgo de infecciones y la presencia de comorbilidades que se suman a la carga de la enfermedad.

Las ponencias seleccionadas abarcan desde los aspectos fisiopatológicos y diagnósticos hasta el manejo terapéutico y el seguimiento clínico de las personas con EM. La selección de ponencias se realizó siguiendo un enfoque temático coherente con el utilizado en ediciones previas de las jornadas Post-ECTRIMS y en los artículos publicados desde su inicio, con el objetivo de garantizar una cobertura equilibrada y continuada de las líneas de investigación con mayor impacto potencial en la práctica clínica.

## 2. Neurodegeneración y Progresión

### 2.1 Una Visión Desde la Patología de las Enfermedades Desmielinizantes

Existe amplia evidencia del papel de las células mieloides en la encefalomielitis autoinmune experimental (EAE), mientras que en la EM es más discutible. El modelo de cuprizona muestra un curso monofásico recurrente, que la inflamación persiste, aunque con diferente evolución, cuando se retira el estímulo tóxico y que la capacidad de remielinización es difícil [1]. En EM, la distribución de células mieloides varía según la evolución de la lesión, desde el centro en lesiones activas hasta la periferia en lesiones mixtas, desapareciendo en lesiones inactivas, mientras que las células T y B persisten en fases posteriores de la lesión [2].

El uso de una puntuación de remielinización muestra que las lesiones mixtas (lesiones crónicas activas e inactivas) son las menos remielinizadas, y que no hay diferencias en los subtipos mieloides entre lesiones activas [3]. La transcriptómica indica cambios tempranos en la sustancia blanca de apariencia normal (SBAN) asociados al desarrollo de lesiones, con diferencias en la expresión génicas de macrófagos y microglía [4]. Cada fase de la lesión presenta un subconjunto mieloide distinto, a su vez distinto del de la SBAN, con macrófagos proinflamatorios que expresan inducible nitric oxide synthase (iNOS) predominantes en la periferia, y oligodendrocitos que disminuyen hacia la periplaca hasta desaparecer en el centro [3, 5]. Aunque algunas lesiones desmielinizantes pueden remielinizarse, en la mayoría de los casos la inflamación persiste en la periferia.

Las principales diferencias en la patología de la EM, el trastorno del espectro de la neuromielitis óptica (NMOSD) y la enfermedad de anticuerpos anti-MOG (MOGAD) se presentan en la Tabla 1 [6]. La neuromielitis óptica (NMO) se caracteriza por el daño al astrocito mediado por complemento, mientras que MOGAD es un proceso de desmielinización mediada por anticuerpos, y en ninguno de los casos hay lesiones crónicas.

**Tabla 1.** **Diferencias en la patología de EM, NMOSD y MOGAD**.

NMO	MOGAD	EM
Predominio CD4^+^	Predominio CD4^+^	Predominio CD8^+^
Astrocitopatía primaria	Desmielinización primaria	Inflamación/desmielinización/microglía
Vénulas pequeñas	Vénulas pequeñas	Venas grandes (SVC)
Lesiones grandes y confluentes en SG y SB	Lesiones grandes y confluentes en SG y SB	
Sólo lesiones activas	Sólo lesiones activas	Lesión crónica activa
No SELs	No SELs	SELs
No PRLs	No PRLs	PRLs
Citotoxicidad celular dependiente del complemento	Citotoxicidad celular dependiente de anticuerpos	

EM, esclerosis múltiple; NMO, neuromielitis óptica; MOGAD, enfermedad de anticuerpos anti-MOG; PRLs, lesiones con borde paramagnético; SB, sustancia blanca; SG, sustancia gris; SELs, lesiones de expansión lenta (del inglés *slowly expanding lesions*); SVC, signo de la vena central; NMOSD, trastorno del espectro de la neuromielitis óptica. Resumen de lo presentado en Comité Europeo para el Tratamiento y la Investigación de la Esclerosis Múltiple (ECTRIMS) 2024 por Hans Lassmann [6].

### 2.2 Mecanismos de Neurodegeneración y su Relación con la Progresión

El suministro metabólico a la neurona y oligodendrocito se deteriora en la EM. Por ello, es importante acompañar las terapias que promueven la remielinización de estrategias que aumenten el suministro energético, siendo la glucosa el principal suministro de energía en el cerebro.

La glucólisis, principal fuente de energía celular, depende de enzimas específicas como la PFKB3, cuya expresión es alta en astrocitos y oligodendrocitos. Las neuronas, en cambio, obtienen energía a través de transportadores de lactato y acetonas desde los astrocitos. El transportador de monocarboxilatos del inglés monocarboxylate transporter 2 (MTC2), clave para el soporte metabólico, está presente en neuronas y oligodendrocitos. Sin embargo, en pacientes con formas progresivas, su expresión en la oligodendroglía está reducida respecto a controles sanos. Modelos experimentales murinos con deleción de MTC2 muestran que la falta de este transportador afecta la mielinización y la oligodendrogénesis.

Los astrocitos tienen capacidad para recordar interacciones inmunes previas, y la histona acetiltransferasa p300 regula esta memoria inmune [7]. Su inactivación en astrocitos *in vivo* mejora la EAE, disminuye la acetilación de histonas e interfiere en la adquisición de la memoria de los astrocitos. P300 utiliza acetil-coenzima A, generada por acetil sintetasa 2 y ATP citrato liasa (Acly), dependiente del metabolismo mitocondrial. La inactivación de Acly también mejora la EAE y reduce la inducción de astrocitos de memoria. Por tanto, la memoria inmune de los astrocitos parece estar controlada por un programa epigenético que depende de Acly y p300. En la EAE [8] y en lesiones crónicas de EM [5], se identifican clústeres de astrocitos con una elevada expresión Acly^+^Ep300^+^. Además, el gen *CLEC16A* se ha identificado como candidato supresor de la actividad proinflamatoria de los astrocitos, y los polimorfismos en este gen se asocian con un mayor riesgo de EM; su inactivación en astrocitos empeora la EAE y provoca la expansión de astrocitos Acly^+^Ep300^+^ (Kadowaki *et al*. *In press*).

### 2.3 PIRA: de los Mecanismos a los Ensayos 

La atrofia de sustancia gris (SG) y medular centran los mecanismos patológicos de la progresión independiente de brotes (PIRA) [9]. Existe una relación entre la atrofia de SG, la discapacidad y PIRA, con mayor afectación en pacientes con progresión silente que en aquellos con formas en brotes, mostrando similitudes con las formas secundariamente progresivas (SP). Se observa un patrón de afectación en lóbulos frontales, parietales y temporales.

La atrofia medular cervical sigue un patrón según el fenotipo: segmentos superiores (C1–C2) en formas tempranas y segmentos inferiores (C6–C7) en formas progresivas [10]. Estudios a largo plazo indican que la atrofia cervical predice el pronóstico y adelanta el diagnóstico de formas progresivas hasta 4 años [11].

Un mínimo de 7 lesiones corticales aumenta la probabilidad de conversión a una forma SP en 6,5 años [12] aunque un estudio reciente no encuentra relación con el PIRA, probablemente por un seguimiento limitado [13]. Parece existir un patrón de desmielinización cortical y talámico, especialmente acusado en formas progresivas, que sugiere factores en líquido cefalorraquídeo (LCR) involucrados, y que se confirma en un estudio con inteligencia artificial (IA) [14].

Las lesiones crónicas activas pueden surgir de la inflamación compartimentalizada, visible mediante resonancia magnética (RM) con lesiones con borde paramagnético (PRLs) y de expansión lenta (SELs), que son parcialmente equivalentes [15, 16]. En el borde de estas lesiones predominan células T, plasmablastos, y una microglía inflamada (MIMS), cargada de hierro, que son anillos magnéticos relacionados con daño inflamatorio y niveles elevados de quitinasa 3-like 1 [17]. Las PRLs y SELs correlacionan con PIRA y discapacidad, y su volumen y carga predicen progresión y severidad a largo plazo [5, 18, 19].

Generalmente, el PIRA no responde al tratamiento, y las terapias de alta eficacia ofrecen un beneficio limitado [20], lo que sugiere una patología subyacente resistente al tratamiento.

## 3. Pródromo y Diagnóstico

### 3.1 EM Prodrómica y Síndrome Radiológico Aislado

Aunque históricamente se ha asumido que la EM no tenía fase prodrómica, distintos estudios muestran un incremento del uso de recursos sanitarios en los pacientes con EM en los 5 años previos a un primer evento desmielinizante [21], que también ocurre en población pediátrica [22].

Los síntomas del pródromo son muy variados y ninguno es específico de la EM. La existencia de un pródromo tiene implicaciones para la prevención secundaria y podría ser importante para evitar el sobrediagnóstico [23, 24, 25]. En prevención primaria, los síntomas prodrómicos deben considerarse en la búsqueda de factores de riesgo de la enfermedad.

Para poder unir el concepto de fase prodrómica con el síndrome radiológico aislado (RIS) y responder a la pregunta de si un RIS es la fase asintomática de la enfermedad, es preciso recalcar que todos los pacientes diagnosticados de RIS acabarán desarrollando una EM con el tiempo [26].

El diagnóstico de RIS puede ser incidental, o deberse a situaciones relacionadas con posibles manifestaciones de la fase prodrómica, como cefalea, o situaciones más desafiantes como síntomas neurológicos visuales transitorios/inexplicables [27]. Sin embargo, la razón para realizar una primera RM no influye en el riesgo de conversión a EM [28]. Además, en la exploración neurológica ante una RM con lesiones sugestivas de EM, los pacientes no refieren quejas sintomáticas [29]. Los pacientes con RIS tampoco muestran un mayor uso de recursos sanitarios [30].

**Fig. 1.**
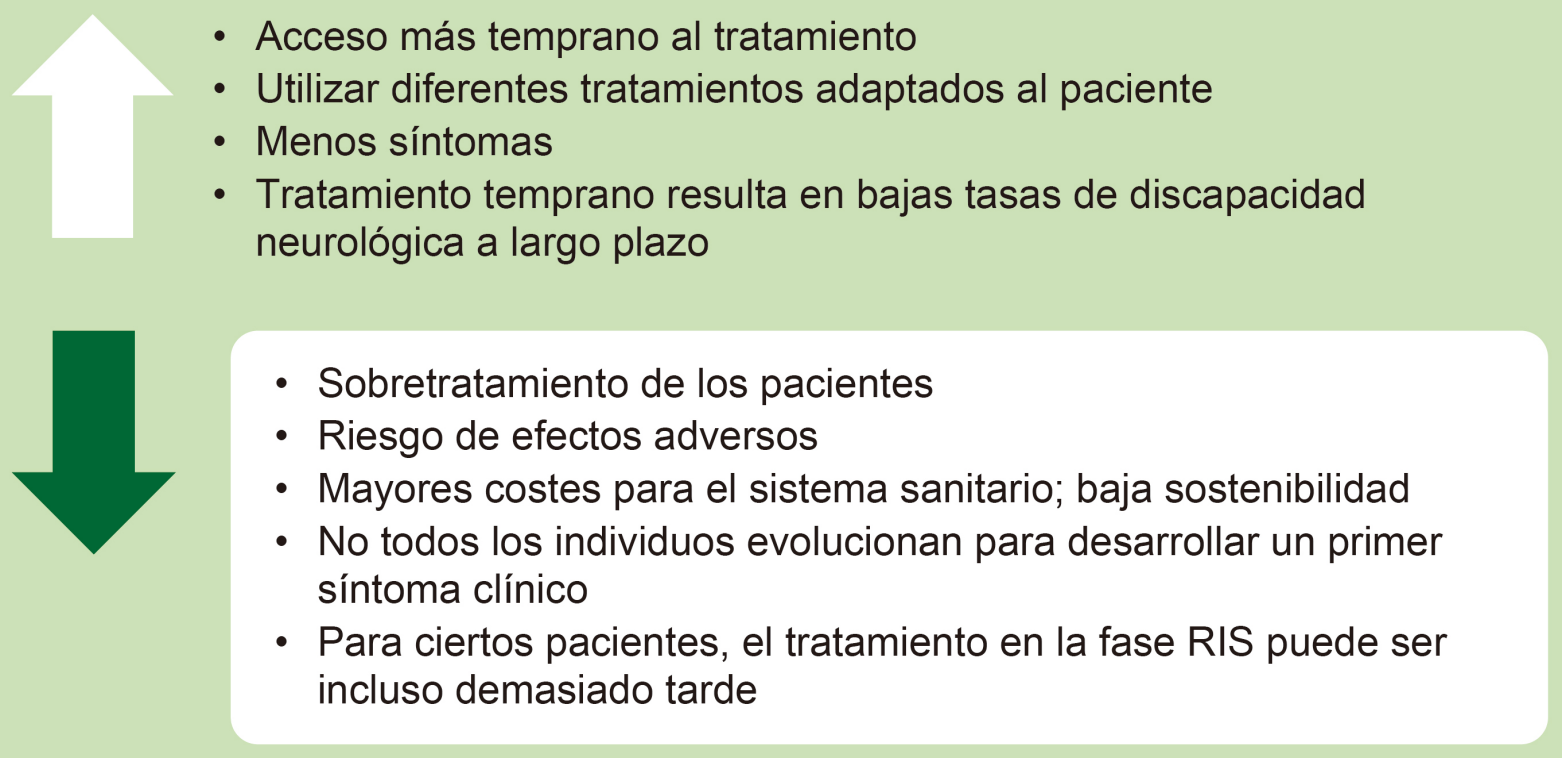
**Ventajas e inconvenientes de no hacer un diagnóstico radiológico correcto y asumir que un RIS es una EM**. RIS, síndrome radiológico aislado. Esta figura fue creada utilizando la versión oficial de Adobe Illustrator 2024 (Adobe Inc., San José, CA, EE. UU.).

En el RIS es frecuente el deterioro cognitivo [31] y también la alteración de la función olfativa [32], de ahí la importancia de utilizar herramientas de valoración sensibles. Se recuerda que una puntuación escala expandida del estado de discapacidad (EDSS) 0 no es neurológicamente normal, habiendo déficits por debajo del umbral en pacientes con dicha puntuación [33]. Las ventajas y los inconvenientes de no hacer un diagnóstico radiológico correcto y asumir que un RIS es una EM se presentan en la Fig. 1. Por último, se nos anima a desplazar nuestra atención a fases más tempranas de evolución de la enfermedad [34].

### 3.2 Nuevos Criterios Diagnósticos de McDonald

**Fig. 2.**
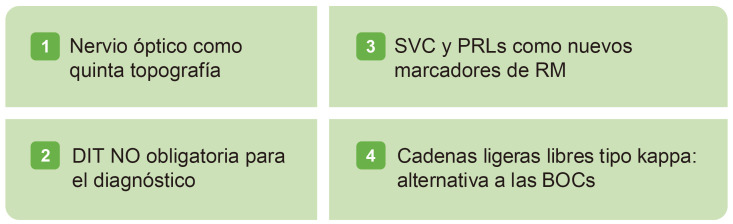
**Novedades de la nueva actualización de los criterios diagnósticos de McDonald**. BOC, bandas oligoclonales; DIT, diseminación en tiempo; PRLs, lesiones con borde paramagnético; RM, resonancia magnética; SVC, signo de la vena central. Presentado en ECTRIMS 2024 por Montalbán y cols [35]. Esta figura fue creada utilizando la versión oficial de Adobe Illustrator.

Se presentaron los nuevos criterios diagnósticos de McDonald [35]. La nueva actualización desde 2017 incluye el nervio óptico como quinta topografía (Fig. 2, Ref. [35]). Lo aprendido en estos últimos años, es que en un primer evento desmielinizante como el síndrome clínicamente aislado (CIS, por sus siglas en inglés), la inclusión de la afectación del nervio óptico aumenta la sensibilidad diagnóstica de una EM clínicamente definida, con una ligera pérdida de especificidad respecto a la diseminación en espacio (DIS) del 2017 [36, 37].

Se añaden el signo de la vena central (SVC) y las PRLs, que aumentan la especificidad. El rendimiento diagnóstico basado en el umbral óptimo para la discriminación del 35% de lesiones SVC [38], es equivalente a la regla “selecciona-6”, más factible para la práctica clínica, definida como la presencia de ≥6 lesiones SVC o si <6 lesiones SVC, que el número de lesiones SCV sea mayor que sin SVC. La presencia de ≥1 PRL muestra una especificidad de un 93% para el diagnóstico de EM [39].

La diseminación en tiempo (DIT) ya no es obligatoria para el diagnóstico, con la evidencia fundamentada en los ensayos clínicos de CIS, y las cadenas ligeras libres Kappa (k-FLC) son una alternativa a las bandas oligoclonales (BOCs) al mostrar una precisión diagnóstica similar, y una medición más sencilla y económica [40].

**Fig. 3.**
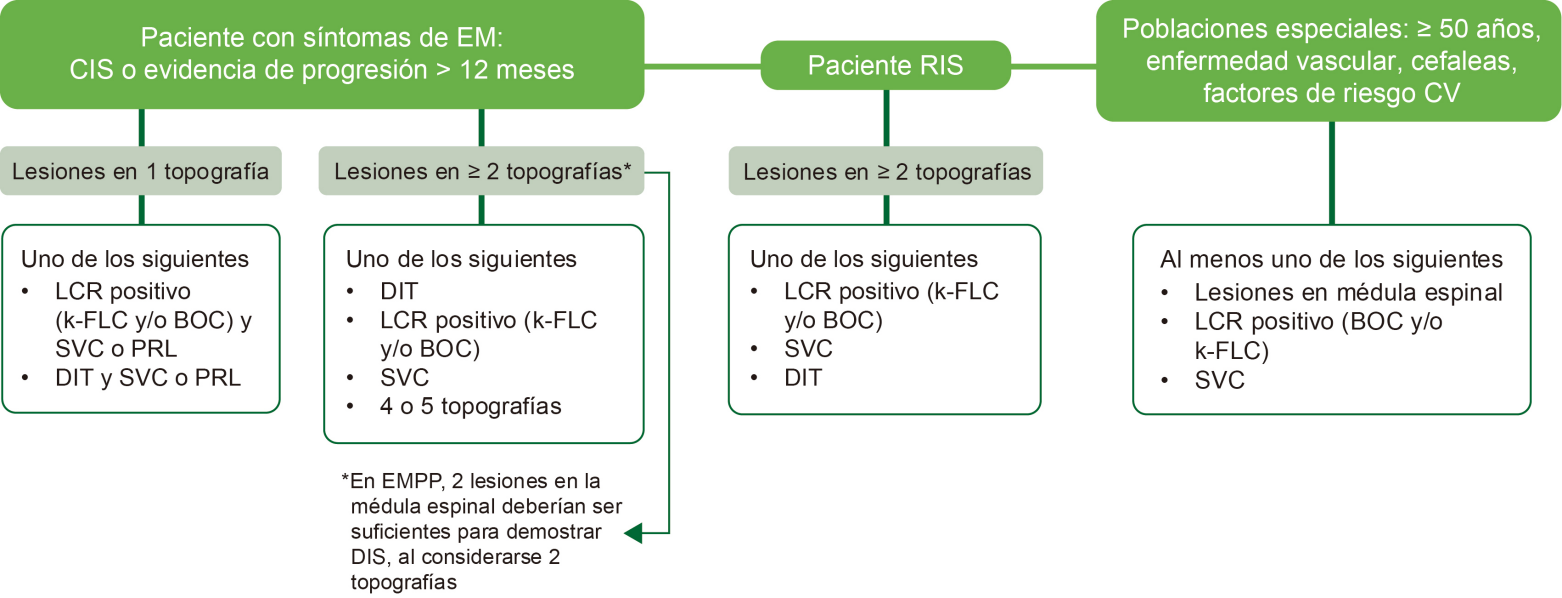
**Algoritmo de aplicación de los nuevos criterios diagnósticos de McDonald**. CIS, síndrome clínicamente aislado; CV, cardiovascular; DIS, diseminación en espacio; EMPP, esclerosis múltiple primariamente progresiva; k-FLC, cadenas ligeras libres tipo kappa; LCR, líquido cefalorraquídeo; PRLs, lesiones con borde paramagnético; RIS, síndrome radiológico aislado. Presentado en ECTRIMS 2024 por Montalbán y cols [35]. Esta figura fue creada utilizando la versión oficial de Adobe Illustrator.

La Fig. 3 (Ref. [35]) representa el algoritmo para la aplicación de los nuevos criterios.

## 4. Utilidad Clínica de Biomarcadores y de la Neuroimagen 

### 4.1 Biomarcadores Útiles en la Práctica

El avance más notable es que la proteína acídica fibrilar glial (GFAP) y los neurofilamentos de cadena ligera (NfLs) son biomarcadores útiles para el seguimiento y estudio de la EM. Iniciativas recientes (*BioMS-eu collaboration*) confirman niveles elevados de GFAP y NfL en formas SP y primariamente progresivas (PP) destacando que la GFAP muestra mejor correlación con la discapacidad, especialmente en pacientes jóvenes con formas SP y EDSS elevada.

Los niveles de NfL deben ajustarse por edad e índice de masa corporal (IMC) para evitar errores. El Z-score, que corrige por estos factores de confusión, mejora la capacidad predictora de brotes en comparación con el umbral de 10 pg/mL, especialmente en jóvenes [41], pero es importante ajustar por encima de 40 años. También será importante ajustar con un Z-score con la GFAP y una plataforma en desarrollo permitirá ajustar los valores de GFAP según edad, sexo e IMC.

Los niveles de GFAP aumentan en pacientes con PIRA, mientras que los NfL son más bajos en aquellos sin PIRA (Benkert *et al*. Ann Neurol *in press*). Además, una reducción de un punto en el Z-score de GFAP reduce un 44% el riesgo de progresión a dos años. La elevación de GFAP, pero no de NfL, en pacientes con PIRA se muestra independientemente del tratamiento, como se observa en cohortes con fingolimod [42] y otros tratamientos [43]. La GFAP se perfila como un marcador más específico de progresión.

La correlación entre los niveles de NfL y el grosor de la médula cervical (ambos marcadores de mal pronóstico) obtuvo resultados algo controvertidos ya que se observaron niveles elevados correlacionando tanto en grosores medulares elevados como disminuidos [44]. Por otra parte, se observó que tanto NfL y GFAP, y grosor medular cervical, fueron marcadores independientes de progresión, lo que sugiere mecanismos de progresión diferentes.

### 4.2 Uso Clínico de la Neuroimagen

La revisión de los marcadores de imagen [45] evidencia que las PRLs se describen en formas progresivas y que un mínimo de 4 PRLs se asocia a un curso más agresivo, brotes clínicos y mayor discapacidad a 10 años. Las SELs indican progresión de la discapacidad y el marcador PET-TSPO podría diferenciar lesiones dudosas y detectar activación glial en SBAN y corteza.

La inflamación leptomeníngea es más visible con imágenes 7T que 3T, pero no se relaciona claramente con lesiones corticales ni es específica de la EM. El aumento de los plexos coroideos tiene baja sensibilidad (55%) pero podría predecir discapacidad a 5 años y ser más una causa de neuroinflamación que una consecuencia. El PET de densidad sináptica aún no es útil en EM.

La tomografía de coherencia óptica (OCT) es clave en el diagnóstico diferencial entre EM, NMO y MOGAD [46]. En EM, el daño retiniano es independiente de la neuritis óptica (NO), siendo mayor en la NMO que en EM. En una NO aguda, el grosor de la capa de fibras nerviosas de la retina ayuda a diferenciar la EM de MOGAD.

Para valorar la utilidad de la neuroimagen en la evaluación de la respuesta terapéutica [47] hay que preguntarse qué consideramos respuesta o fallo al tratamiento. Mantener el estado NEDA (del inglés, no evidence of disease activity) es complicado y su ausencia no implica acúmulo de discapacidad. También es sabido que la RM es 5–10 veces más sensible que la clínica para detectar actividad [48], y quizás lo más importante sea analizar si los sistemas de puntuación (Río, canadiense, etc.), son útiles con los fármacos de alta eficacia, ya que teóricamente la evolución esperada con estos fármacos no es la real [49].

## 5. Resultados Informados por los Pacientes y Monitorización Digital

### 5.1 Perspectivas de las Medidas de Resultados Informados por los Pacientes 

Cabe reflexionar sobre si la obsesión con medir y cuantificar el rendimiento humano a todos los niveles, y completar datos de registros y bases de datos, verdaderamente ayuda a los pacientes [50]. Solo deberían aplicarse medidas útiles para la toma de decisiones junto con el paciente, cuestionando qué se mide, por qué y cómo se utilizará esa información. Un problema relacionado es la falta de interés que indican haber sufrido el 88% de los pacientes por parte de sus médicos que desatienden, menosprecian o niegan sus preocupaciones. Es importante educar a los pacientes sobre cómo la automonitorización a través de las medidas de resultados informados por los pacientes (PROs) les permite tener una mayor información sobre su enfermedad, promueve cambios en su estilo de vida y les guía en la preparación de sus visitas médicas.

La selección de PROs en los ensayos clínicos no se basa en la evidencia, sino que es aleatoria, lo que genera una falta de consistencia que impide comparar datos. Una revisión reciente de 21 protocolos concluye que ninguno describía la estrategia de selección de los instrumentos utilizados, y recomienda justificar la elección y comparar el rendimiento entre los PROs seleccionados [51].

En EE. UU., iniciativas como PROMIS [52] y NeuroQoL [53] utilizan la “teoría de respuesta al ítem” para desarrollar PROs más breves, precisos y adaptativos. Este modelo psicométrico analiza cada ítem individualmente, y crea una curva de respuesta para cada ítem a partir de datos de una muestra representativa de la población diana. Mediante esta metodología, se han construido librerías de ítems de todas las dimensiones y dominios de la salud. En EM, el ejemplo lo tenemos con las iniciativas PROMS y del grupo de trabajo de EM del consorcio Clinical Path Institute.

### 5.2 Implicaciones Clínicas de la Monitorización Digital y la Inteligencia Artificial 

Los resultados de un registro del Reino Unido iniciado en 2021, con un millón de instrumentos digitalizados cumplimentados hasta la fecha, incluyendo la EDSS digital (UK MS Register), muestra que no hay diferencias entre los datos recopilados de manera electrónica y los obtenidos como parte de un ensayo clínico. Un algoritmo desarrollado para identificar casos de EM del banco de datos SAIL, ha demostrado una sensibilidad diagnóstica del 96,7% [54].

La batería online Cognitron, diseñada para entrenar y mejorar la función cognitiva, está en fase de validación. De las 22 pruebas iniciales, 12 fueron seleccionadas por su mayor sensibilidad para detectar una disminución del rendimiento cognitivo, y menor redundancia y sensibilidad al dispositivo. Estas se completaron con y sin supervisión neuropsicológica, mostrando una correlación moderada [55].

Por su parte, el programa RADAR-CNS explora dispositivos portátiles y teléfonos inteligentes para monitorizar enfermedades del sistema nervioso central. En EM, un estudio prospectivo integró medidas estándar como la EDSS, la prueba de la marcha de los 6 minutos (6MWT) y la prueba de la marcha de 25 pies (T25FW), además de monitorización remota con un dispositivo portátil (Fitbit). Los resultados mostraron que los pacientes con progresión confirmada de la discapacidad (según EDSS) dieron menos pasos que aquellos sin progresión, pero la disminución de pasos a lo largo del tiempo fue similar en ambos grupos [56]. Resultados similares se observaron con 6MWT y T25FW, sugiriendo que medidas actuales como EDSS podrían no detectar cambios funcionales sutiles.

Un desafío en el uso clínico de dispositivos portátiles es la baja participación de los pacientes, que limita la transferencia de datos, especialmente en medidas como frecuencia cardíaca, sueño o conectividad, aunque los datos pasivos se recopilan de forma consistente.

## 6. Infecciones: Patogénesis, Riesgo y Prevención

### 6.1 Virus de Epstein-Barr y EM

El mimetismo molecular de alta afinidad entre EBNA-1 y proteínas como GlialCAM [57], anoctamina 2 [58], o alfa-cristalina [59] parece ser responsable de la autoinmunidad cruzada. La respuesta de anticuerpos al EBNA-1 es el principal factor de riesgo serológico para la EM [60].

Un déficit en la expresión génica causa anomalías en la respuesta inmunológica al virus de Epstein-Barr (VEB) en pacientes con EM; la expresión débil de NKG2D es un posible mecanismo subyacente a la vulnerabilidad al VEB [61]. Por otro lado, la constante mutación del virus expone vulnerabilidades que podrían aprovecharse para el diseño de terapias antivirales innovadoras [62]. Iniciativas de mapeo genético del virus buscan entender mejor su interacción con la EM.

### 6.2 Riesgo de Infecciones y Vacunación

La incidencia de infecciones aumenta en el año previo al diagnóstico [63] y durante el curso de la enfermedad, sobre todo en cursos progresivos y de elevada discapacidad [64].

Con el tratamiento anti-CD20, existe un riesgo de reactivación del virus de la hepatitis B [65], aunque en muy raras ocasiones [66], y un mayor riesgo de hospitalización y uso de antibióticos en comparación con los fármacos plataforma [67]. El riesgo de infecciones tratadas en el hospital parece aumentar con el tiempo de exposición al fármaco [68].

La vacunación es segura y no aumenta el riesgo de hospitalización por brotes [69], y el primer consenso europeo sobre vacunación en EM [70] recomienda vacunar al diagnóstico o en las primeras fases de la enfermedad.

Excepto los anti-CD20 y moduladores S1P, los tratamientos modificadores de la enfermedad (TME) no modifican la respuesta humoral a la mayoría de las vacunas [71, 72, 73]. Las terapias anti-CD20 no comprometen la inmunidad preexistente frente al virus de la varicela zóster o el sarampión, aunque los títulos basales más bajos de IgG se asocian a un mayor riesgo de pérdida de seroprotección [74].

## 7. Comorbilidad en EM

La tasa de comorbilidad en EM es elevada desde el diagnóstico [75] y aumenta con el tiempo. A diferencia de los trastornos de ansiedad y depresión, la comorbilidad cardiovascular aumenta con la edad.

**Fig. 4.**
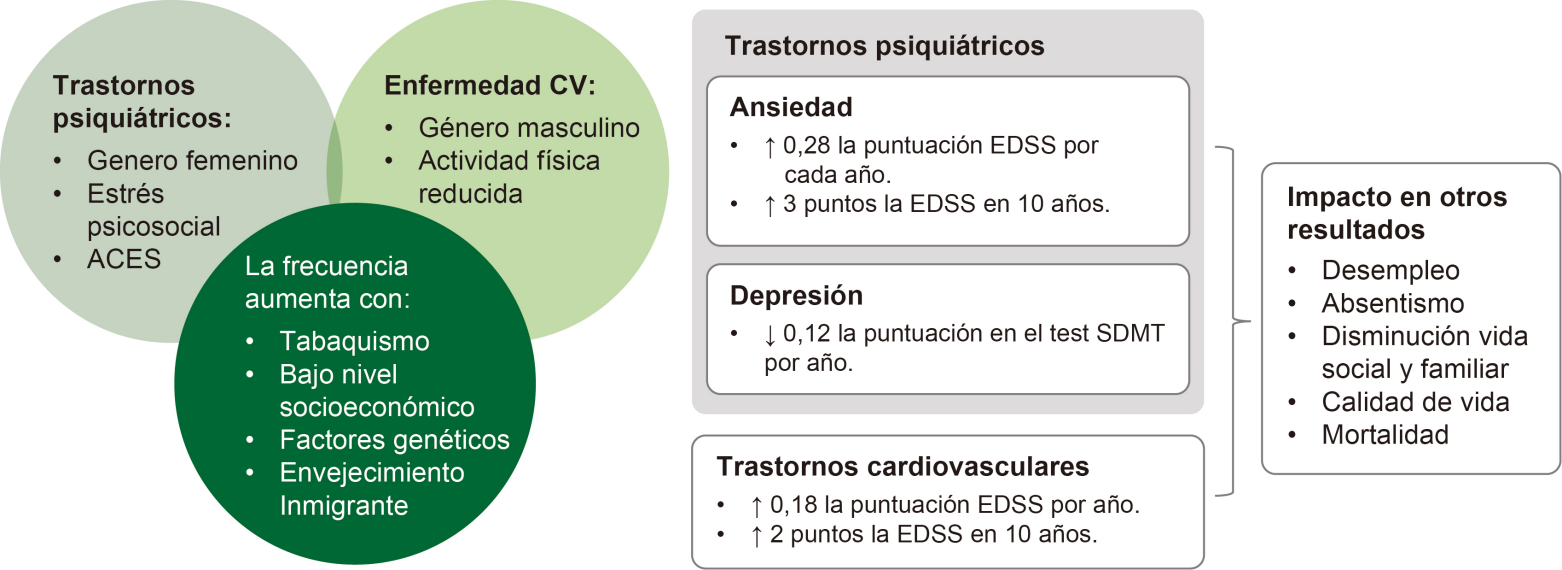
**Diferencias poblacionales y de resultados según la comorbilidad**. ACES, experiencias adversas en la infancia; EDSS, escala expandida del estado de discapacidad; SDMT, test de símbolos y dígitos (del inglés symbol digt modalities test). Las flechas indican la dirección del cambio observado: ↑ representa un aumento o incremento en el parámetro medido; ↓ representa una disminución o reducción en el parámetro medido. Esta figura fue creada utilizando la versión oficial de Adobe Illustrator.

En función de la comorbilidad, se observan diferencias poblacionales [76, 77, 78], de progresión de la discapacidad [79, 80] y de velocidad de procesamiento de la información [81] (Fig. 4). Esto es importante puesto que la carga de comorbilidad de los participantes en ensayos clínicos puede afectar los resultados, como muestra el estudio CombiRX [82].

El impacto clínico de la comorbilidad en los brotes, deterioro físico y cognitivo, y estilo de vida es indiscutible, y se recuerda la importancia de educar al paciente en hábitos saludables. El manejo de las comorbilidades debe integrarse en la atención de la EM; es decir, hay que tratar la EM considerando las comorbilidades y tratar las comorbilidades considerando la EM [83], y por supuesto individualizarse.

Los pacientes con NMO también experimentan una carga de enfermedad agravada por las comorbilidades, presentes en el 66,7% de los pacientes [84]. Estudios genéticos han identificado genes de susceptibilidad, incluidas asociaciones compartidas entre enfermedades autoinmunes clínicamente distintas [85], y una relación causal entre el lupus eritematoso sistémico (LES) y el síndrome de Sjögren con la NMO, pero no al revés [86]. Hasta un 25% de los pacientes con una enfermedad autoinmune puede desarrollar otra; el registro danés con datos de más de 2 millones de mujeres y 22 enfermedades autoinmunes muestra grupos de enfermedades autoinmunes reumatológicas, neurológicas y gastrointestinales que tienden a coincidir [87]. Otro dato interesante es que más del 80% de los pacientes con NMO y poli-autoinmunidad (LES, Sjögren o miastenia grave) presentan anticuerpos IgG anti-acuaporina 4 [88]. En MOGAD, sin embargo, es poco común la autoinmunidad asociada [89].

## 8. Conclusiones

En la edición post-ECTRIMS 2024 se presentaron varios avances en la investigación de EM. En lesiones activas de EM no hay correlación entre el fenotipo de célula mieloide y la remielinización, aunque en modelos animales ciertos subtipos de células inmunes muestran un efecto beneficioso sobre oligodendrocitos y remielinización.

Las terapias remielinizadoras deben superar el ambiente inflamatorio hostil, y acompañarse de estrategias que aumenten el suministro energético, siendo los monocarboxilatos fundamentales como soporte metabólico para las neuronas y oligodendrocitos. También se ha demostrado que los astrocitos tienen capacidad para recordar interacciones inmunes previas dando lugar a astrocitos de memoria, que están incrementados en las lesiones crónicas activas y regulados por el gen *CLEC16A*. La atrofia de SG se relaciona con la discapacidad y el PIRA, con mayor afectación en pacientes con progresión silente que en aquellos con formas en brotes. Por su parte, la atrofia medular cervical sigue un patrón según el fenotipo, predice el pronóstico y permite adelantar el diagnóstico de formas progresivas hasta 4 años.

Un dato importante a tener en cuenta es el aumento del uso de recursos sanitarios de los pacientes con EM en los años previos a un primer evento desmielinizante. Los síntomas prodrómicos son muy variados y ninguno es específico de la EM, pero pueden ser claves para identificar factores de riesgo de la enfermedad, y se recuerda que el diagnóstico del RIS también puede deberse a situaciones relacionadas con manifestaciones de la fase prodrómica. En esta línea, los nuevos criterios de McDonald permitirán diagnosticar EM en algunos pacientes que se clasificarían como RIS, y la diseminación en tiempo ya no debería ser necesaria para diagnosticar la EM.

Otro avance notable es la utilidad de los biomarcadores GFAP y NfLs para el seguimiento y estudio de la EM, mientras que PRLs, SELs y PET-TSPO emergen como prometedores marcadores de imagen de lesiones crónicas activas.

En otra línea, se instó a que la valoración de los datos en salud informados por los pacientes se realice con herramientas verdaderamente útiles, breves y adaptativas; la digitalización de estas herramientas muestra potencial para complementar las medidas estándar, aunque enfrenta desafíos en la transferencia de datos debido a la baja participación de los pacientes.

En cuanto a las infecciones, se muestra que hay un aumento del riesgo evidente desde el año previo al diagnóstico, que puede exacerbarse con ciertos tratamientos. Asimismo, se pone de manifiesto la elevada tasa de comorbilidades al diagnóstico y el impacto clínico, y se recomienda integrar su manejo como parte fundamental de la atención en EM.

## Contribuciones de los Autores

Todos los autores del Grupo Post-ECTRIMS han contribuido por igual en la elaboración de este artículo. OF, AArés, EA, YA, AAlonso, RA, LB, CC, ABC, TCT, LCF, SE, MAH, LL, ML, SL, JML, VML, EM, COG, JMP, LRP, AV, ARA han contribuciones sustanciales a la concepción o diseño de la obra, participado en la redacción del manuscrito o en su revisión crítica por contenido intelectual relevante, han dado su aprobación final a la versión que será publicada y han aceptado la responsabilidad por todos los aspectos del trabajo, asegurando que cualquier cuestión relacionada con la exactitud o integridad de alguna parte de este sea debidamente investigada y resuelta.

## Aprobación Ética y Consentimiento Informado

No aplicable.

## Agradecimientos

Los autores quisieran mostrar su agradecimiento a Isabel Caballero y Laura Prieto del Val de Evidenze Health España, S.L.U por los servicios de redacción médica prestados en la realización del artículo.

## Financiación

La elaboración de este artículo ha sido financiada por Merck.
